# Case report: Identification of a novel *TOR1AIP2::ETV6* transcript with *FLT3*-ITD mutation in acute myeloid leukemia progressed from myelodysplastic syndrome

**DOI:** 10.3389/fonc.2024.1466590

**Published:** 2024-12-10

**Authors:** Jun Xia, Guanqun Yang, Qingling Yu, Ruiyi Zhang, Ge Li, Man Wang, Hongli Sun, Heng Chen, Lingling Wang, Ping Chen, Kai-Li Gu, Chao Sun

**Affiliations:** ^1^ Department of Hematology, Affiliated Wuxi People’s Hospital, Nanjing Medical University, Wuxi, China; ^2^ Changshu Hospital Affiliated to Soochow University, Changshu NO.1 People’s Hospital, Changshu, Jiangsu, China; ^3^ Department of Hematology, Affiliated Jianhu Hospital of Nantong University Xinglin College, Yancheng, China; ^4^ Suzhou Jsuniwell Medical Laboratory, Suzhou, China; ^5^ Jiangsu Institute of Hematology, National Clinical Research Center for Hematologic Diseases, Key Laboratory of Thrombosis and Hemostasis, The First Affiliated Hospital of Soochow University, Suzhou, China

**Keywords:** acute myeloid leukemia, gene fusions, TOR1AIP2::ETV6, FLT3-ITD, mutation

## Abstract

Acute myeloid leukemia (AML), which is most common in adults, is a challenging hematological malignancy. The occurrence and the progression of AML are often accompanied by various gene fusions and/or mutations. Herein, we report the first case of a *TOR1AIP2::ETV6* fusion transcript with a translocation of *t*(1;12)(q25;p13) in AML progressed from myelodysplastic syndrome (MDS) combined with an *FLT3*-ITD (internal tandem duplication) mutation. Further studies should focus on the biological functions of these novel chimeric products in disease onset and progression, as well as their potential as monitoring markers in disease regression.

## Introduction

Acute myeloid leukemia (AML) is a malignant clonal disease characterized by the impaired proliferation and differentiation of myeloid immature cells, leading to the dysfunction of normal hematopoietic function (1) .There is a lot of evidence showing that the occurrence and the development of AML are often accompanied by the emergence of gene fusions (e.g., *PML::RARA*, *CBFB::MYH11*, and *RUNX1::RUNX1T1*, among others) and mutations such as *c-KIT*, *FLT3*-ITD, *NPM1*, and *TP53* (2, 3). Approximately 20%–30% of newly diagnosed AML patients have an *FLT3*-ITD mutation, which is a representative marker indicating poor overall survival and recurrence of AML (4). These molecular markers provide an extremely important basis for clinical AML diagnosis and treatment, prognosis evaluation, and minimal residual disease (MRD) monitoring (5, 6). Furthermore, the interactions between gene fusions and mutated genes have been reported to be prominent driving factors for the onset of AML (7). With an increasing number of new fusions identified, this provides a promising future for the exploration of pathogenesis and new therapeutic targets for hematopoietic malignancies.

Herein, we report a novel *TOR1AIP2::ETV6* transcript that was predicted to express no fusion protein in a patient with AML that progressed from MDS with the *FLT3*-ITD mutation. Interestingly, the *FLT3*-ITD mutation in the present case was not detected by a next-generation sequencing (NGS) panel, while the chimeric fusion remained detectable after treatment with the venetoclax and azacitidine combined regimen. In addition, the fragments per kilobase million (FPKM) value of the *TRO1AIP2* of this patient appeared to be higher compared with those of other patients with AML, indicating that such fusion transcript could lead to the onset of AML via regulation of the expression of *TRO1AIP2*. Whether this fusion transcript represents a new tumor subclone and acts as a monitoring marker warrant more sequential studies. This case report might provide new insights into understanding the occurrence and progression of AML.

## Case presentation

**Figure 1:**
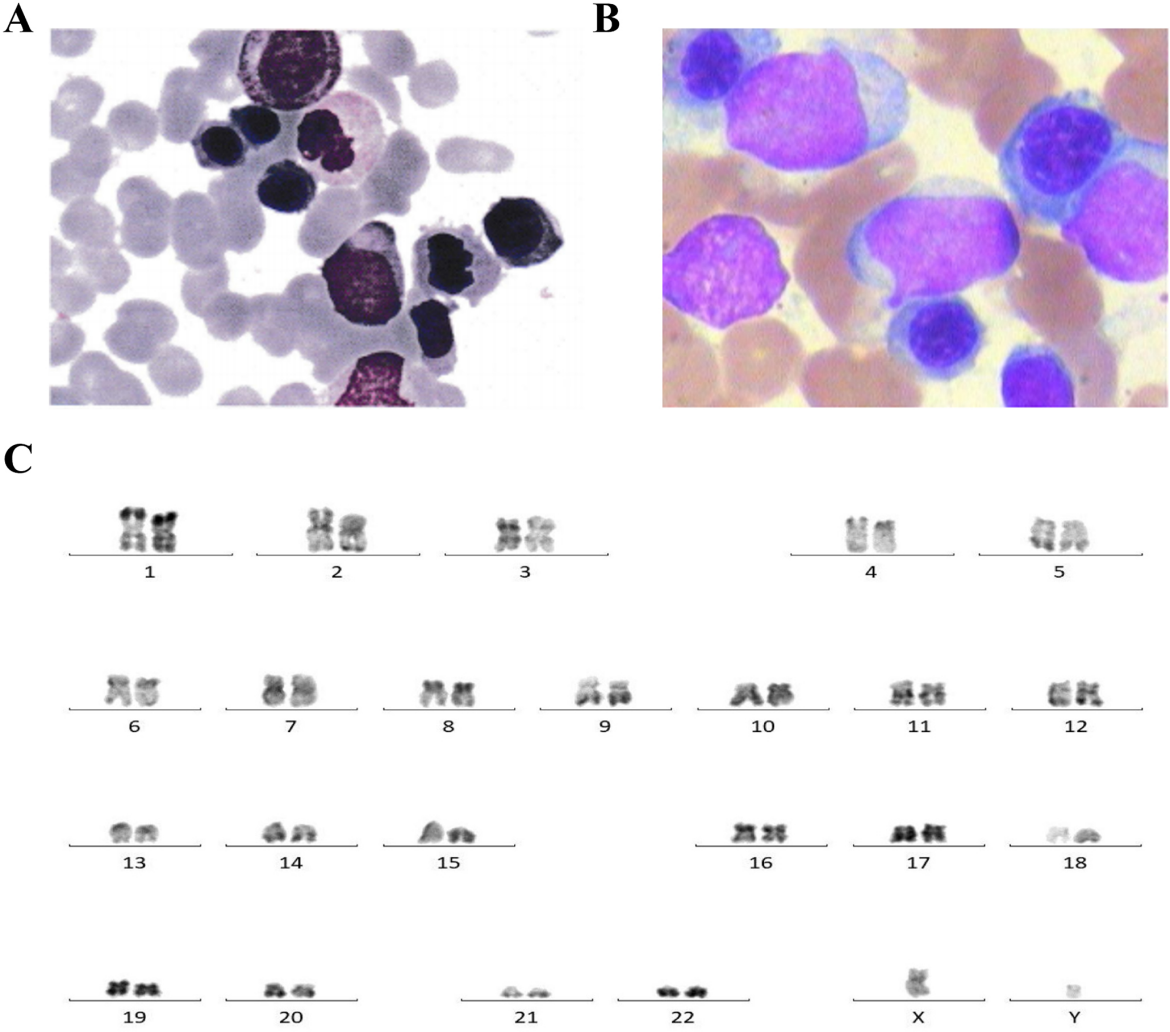
**(A)** Bone marrow cytology showing protoplasmic cells accounting for 10% in this patient for the newly diagnosed myelodysplastic syndrome with excess blasts-2 (MDS-EB2). **(B)** Bone marrow cytology showing primitive cells accounting for 40% in acute myeloid leukemia (AML) progressed from myelodysplastic syndrome (MDS). **(C)** Karyotype analysis showing normal 46,XY [20].

A 72-year-old man was first diagnosed with myelodysplastic syndrome with excess blasts-2 (MDS-EB2) with protoplasmic cells accounting for 10% in 2016 and received multiple rounds of chemotherapy (**Figure 1A**). After admission, a complete routine peripheral blood examination showed a white blood cell count of 0.11 × 10^9^/L, a hemoglobin level of 84.0 g/L, and a platelet count of 125 × 10^9^/L. The albumin, globulin, and C-reactive protein (CRP) levels were 26.8 g/L, 37.8 g/L, and 64.1 mg/L, respectively.

The bone marrow (BM) aspirate smear showed primitive cells accounting for about 40% (**Figure 1B**). The karyotype analysis showed normal karyotype (**Figure 1C**). Flow cytometry analysis of the BM showed residual tumor cells, which expressed CD34, CD7, CD33, and CD38; partially expressed CD117; and were negative for CD13, HLA-DR, CD10, CD11b, CD19, and CD56.

**Figure 2:**
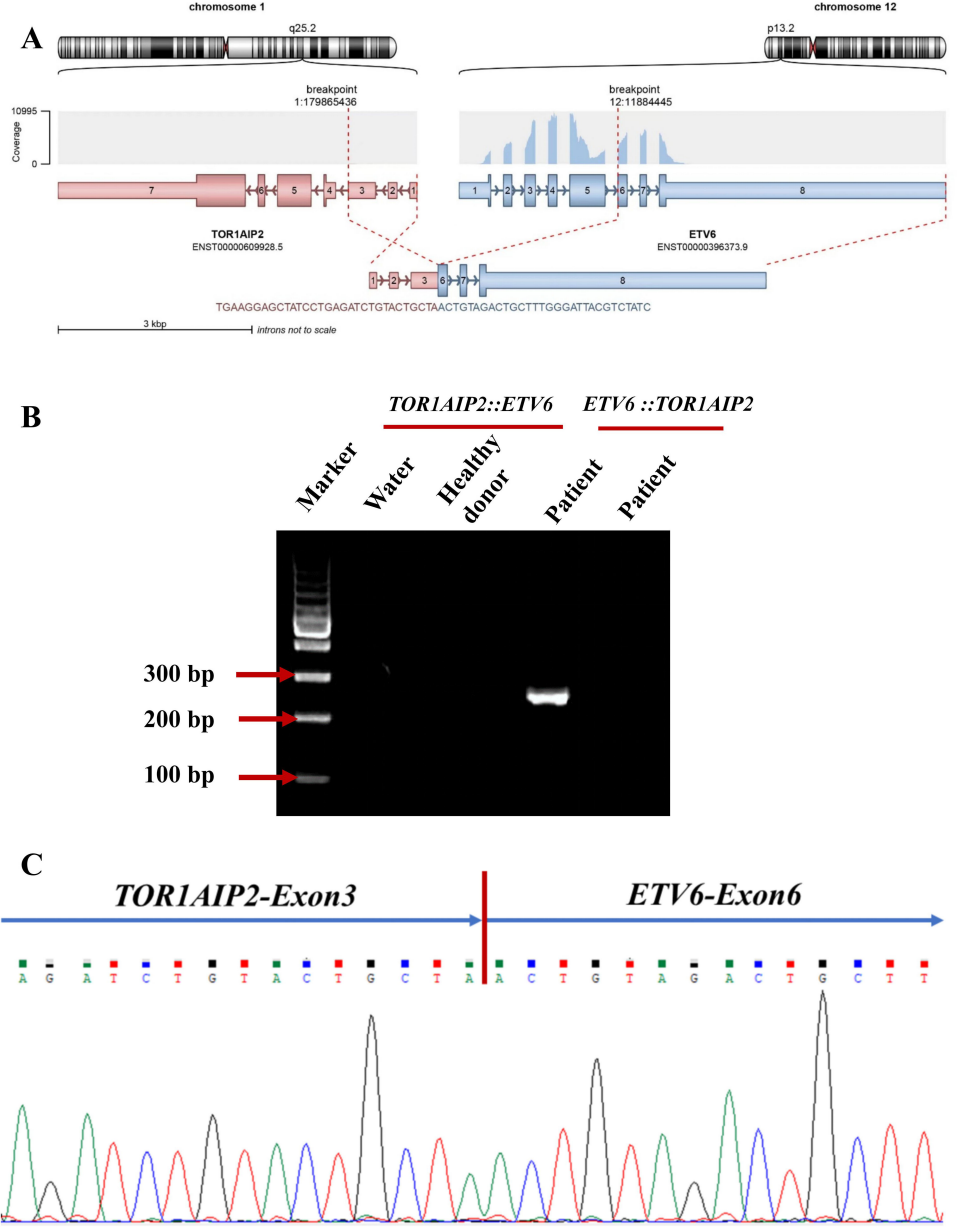
**(A)** Schematic diagram of the formation mechanism of the *TOR1AIP2::ETV6* transcript. **(B)** Electrophoresis of the RT-PCR products from the patient showing the *TOR1AIP2::ETV6* fusion transcript. **(C)** Partial nucleotide sequences surrounding the junctions of the *TOR1AIP2::ETV6* fusion transcript.

Subsequently, NGS was performed using an 82-gene panel to identify gene mutations and a 71-gene panel to carry out a more comprehensive fusion screening through targeted RNA sequencing (RNA-seq). The results showed detection of a 36- and a 123-bp *FLT3*-ITD. At the same time, the *FLT3*-ITD mutations were examined using capillary electrophoresis (CE). The results showed that *FLT3*-ITD had mutated in this patient (**Supplementary Figure S1C**). *NPM1* mutations were not detected. The targeted RNA-seq data were analyzed using STAR-Fusion, which identified a novel fusion transcript of *TOR1AIP2::ETV6*, fused by *TOR1AIP2* (NM_001199260) exon 3 with *ETV6* (NM_001987.5) exon 6 through translocation (**Figure 2A**). To confirm the existence of the chimeric transcript, primers were designed to amplify the fusion product. The forward and reverse primers were TGTCAGCTCCTCTGTTTCACC and AGTTTTCGTACCGGCTGTCA, respectively. Using agarose gel electrophoresis, the targeted fusion product was shown in a band in 249 bp, while the reciprocal fusion *TOR1AIP2::ETV6* was not found (**Figure 2B**). Furthermore, Sanger sequencing was used, which verified the presence of the *TOR1AIP2::ETV6* fusion transcript (**Figure 2C**). Molecular diversity was not assessed at the time of initial diagnosis due to the limited laboratory technology.

**Figure 3:**
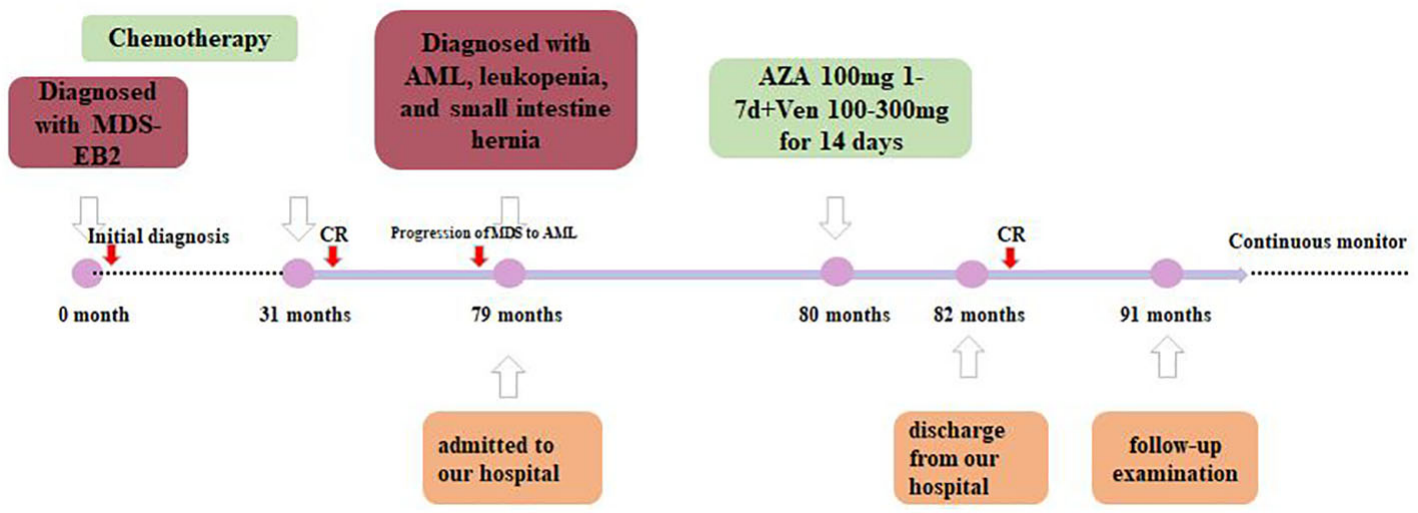
Timeline of therapy.

The patient was diagnosed with progression of MDS to AML according to the 5th World Health Organization (WHO) classification. Since the patient was a 72-year-old elderly person with lower tolerance, the venetoclax plus azacitidine therapy regimen was chosen (100 mg azacitidine for 7 days, then 100–300 mg venetoclax for half a month, and then 100–200 mg venetoclax for maintenance treatment, and no longer with azacitidine) as an individualized dosing regimen for treatment due to his old age and poor physical condition (**Figure 3**). The condition of the patient improved after treatment and he was discharged from our hospital.

A year after the recurrence, the patient came back for a follow-up examination. The primitive cells accounted for 4%. The 11 flow cytometry panels for AML-MRD were conducted, with positive results. A total of 239,980 nucleated cells were detected, of which 2,266 were abnormal, accounting for 0.94% of the nucleated cells (the results are not shown in the figure). In addition, the *FLT3*-ITD mutation was undetectable with NGS and CE using the patient’s BM, with a mutation detection sensitivity of 1%. However, the chimeric fusion transcript was still present, with the relative expression of its transcript being 0.35% by quantitative PCR (qPCR). The timeline of therapy is shown in **Figure 3**.

## Discussion

In the current study, we report the first case of a *TOR1AIP2::ETV6* fusion in a patient with AML that progressed from MDS using targeted RNA-seq, underlining its application in the identification of minor chromosomal variations with elevated sensitivity in spite of the karyotype analysis showing a normal karyotype.

*ETV6*, previously known as *TEL*, is located on chromosome 12p13 and is a member of the ETS (E-Twenty-Six) family of transcription factors. The *ETV6* gene consists of eight exons. The *ETV6* protein is composed of two major domains: the helix–loop–helix (HLH) and ETS domains. The HLH domain is encoded by exons 3 and 4 and functions as a homo-oligomerization domain, while the ETS domain, which is encoded by exons 6–8, is responsible for protein–protein interactions. A central domain, called the internal domain, is involved in the recruitment of a repression complex including *NCOR1*, *NCOR2*, and *SIN3A* (9). The *ETV6* protein and other ETS proteins work together to affect the process of cell proliferation, differentiation, tumorigenesis, and hematopoietic regulation (10). Multiple chromosomal rearrangements involving *ETV6* (such as *ETV6::RUNX1*, *ETV6::ABL1*, *ETV6::NTRK3*, and *ETV6::ACSL6*) have been demonstrated to play a crucial role in the development of several hematologic diseases such as AML, as well as in their diagnosis and prognosis (11). Thus far, 103 gene fusions involving *ETV6* due to translocations, insertions, or inversions have been identified, as shown in the Mitelman database (https://mitelmandatabase.isb-cgc.org-accesssed).

In this paper, we report a brand new fusion partner of *ETV6*, named torsin 1A interacting protein 2 (*TOR1AIP2*), which is a protein-coding gene located at 1q25.2. *TOR1AIP2* is required for endoplasmic reticulum integrity. It regulates the distribution of *TOR1A* between the endoplasmic reticulum and the nuclear envelope and induces the ATPase activity of *TOR1A*, *TOR1B*, and *TOR3A* (12). There are only two *TOR1AIP2* fusion partners reported: *TOR1AIP2::ACBD6* and *TOR1AIP2::IFI16* (13). The *TOR1AIP2::ETV6* transcript is formed through translocation, with *TOR1AIP2* breaking at chr1:179865436:− and *ETV6* breaking at chr12:11884445:+. Despite the confirmation of novel fusion transcripts using different methods, no chimeric fusion protein was predicted due to a stop codon being formed in the junction site, which might generate a truncated *ETV6* protein. It is possible that the production of *ETV6* truncated transcripts could result in a pathogenic role for *ETV6* haploinsufficiency in this patient. In addition, no evidence of the reciprocal *ETV6::TOR1AIP2* fusion transcript was found. This suggests a more complex translocation process, possibly combined with further transcriptional or posttranscriptional regulation. Interestingly, the expression level of *TOR1AIP2* represented by the FPKM of this patient was higher than those of patients with AML without such chimeric fusion, indicating that it could lead to the onset of AML via the upregulation of the expression of *TOR1AIP2*, with the *ETV6* FPKM value remaining unaffected (**Supplementary Figure S1**).

In our case, *FLT3*-ITD was also detected, which occurs in 20%–30% of *de novo* AML patients and is associated with disease relapse, as well as with inferior overall survival (14). In the present study, the patient carried an *FLT3*-ITD with a low MAF of 2% and was negative for *NPM1* mutations, which represent intermediate risk according to the 2022 European Leukemia Network (ELN) (8). Research has shown that the interactions between gene fusions and mutated genes play a crucial role in the prognosis and recurrence of AML (7). Thanasopoulou’s group provided experimental proof that the co-expression of *NUP98::NSD1* and *FLT3*-ITD could collaborate for transformation *in vitro* and the progression of AML *in vivo* (15). Schessl et al. reported that *AML1::ETO* collaborated with *FLT3* length mutation and could induce acute leukemia in a murine BM transplantation model (16). Moreover, *in vivo*, Kim et al. found that *FLT3*-ITD mutations could cooperate with *CBFB::SMMHC* in an animal model of inv(16)-accompanied AML (17). More research should focus on whether the co-expression of the *ETV6::TOR1AIP2* fusion transcript with *FLT3*-ITD could act synergistically in the onset and progression of AML.

*FLT3* inhibitors (including sorafenib and gilteritinib, among others) and allogeneic hematopoietic cell transplantation (alloHCT) are commonly used as treatments for AML with *FLT3*-ITD (18). Short et al. found that the combination of azacitidine, venetoclax, and gilteritinib could improve survival in newly diagnosed *FLT3*-mutated AML (19). After being administered the venetoclax plus azacitidine regimen, the patient was assessed for recurrence. Interestingly, *FLT3*-ITD was no longer detected using the NGS panel, indicating a decrease in the *FLT3*-ITD clones with this treatment regimen. However, the fusion transcript was still present. Longitudinal monitoring of the current patient might illustrate whether the fusion transcript represents a leukemia subclone. Furthermore, the *FLT3*-ITD mutation decreased with a regimen without the *FLT3* inhibitor, indicating the possibility that the combination of the current fusion and *FLT3*-ITD may be more sensitive to chemotherapy.

## Conclusion

In summary, to the best of our knowledge, this is the first report of a *TOR1AIP2::ETV6* transcript co-expressed with the *FLT3-ITD* mutation in AML progressed from MDS. This novel fusion combined with *FLT3*-ITD showed potential as a monitoring marker. We hope that this case report can provide a new perspective for understanding the disease progression of AML, as well as for treatment options for *FLT3*-positive AML patients.

## Data Availability

The datasets presented in this study can be found in online repositories. The names of the repository/repositories and accession number(s) can be found in the article/**Supplementary Material**.
