# Supplementary material for: Case report: Identification of a novel TOR1AIP2::ETV6 transcript with FLT3-ITD mutation in acute myeloid leukemia progressed from myelodysplastic syndrome
Source: Front Oncol. 2024 Dec 10;14:1466590. doi: 10.3389/fonc.2024.1466590 (PMC11669196; doi:10.3389/fonc.2024.1466590)
Supplement: Supplementary file 1 [file DataSheet1.pdf]

Supplementary Appendix

Supplement to: Identification of a novel TOR1AIP2::ETV6 transcript with FLT3-ITD mutation in acute myeloid leukemia progressed from myelodysplastic symdrome.

Myeloid neoplasms and acute leukemia gene mutation panel (82 genes)

ABL1	CEBPA	ETNK1	IL7R	NF1	RAD21	SOS1
ANKRD26	CEBPE	ETV6	JAK1	NOTCH1	RAF1	SRP72
ARID5B	CRLF2	EZH2	JAK2	NPM1	RARA	SRSF2
ASXL1	CSF3R	FAT1	JAK3	NRAS	RB1	STAG2
ASXL2	CSMD1	FBXW7	KANSL1	PAX5	RUNX1	TET2
BCOR	CTCF	FLT3	KDM6A	PDGFRA	SETBP1	TP53
BCORL1	DDX41	GATA1	KIT	PDGFRB	SETD2	U2AF1
BRAF	DHX15	GATA2	KMT2A	PHF6	SF3B1	WT1
CALR	DNMT3A	GATA3	KRAS	PIGA	SH2B3	ZBTB7A
CBL	ECT2L	IDH1	MECOM	PPM1D	SMC1A	ZRSR2
CDKN2A	EED	IDH2	MPL	PTEN	SMC2	
CDKN2B	EP300	IKZF1	MYC	PTPN11	SMC3	

Detection method

1. Probe capture library construction method is used for DNA sequencing, with an average sequencing depth of more than 1000×, and all data quality analysis meets Q30. This report only detects point mutations, small fragment insertion or deletion mutations of the target gene, with a detection sensitivity of 2%.
2. FLT3-ITD is verified by PCR capillary electrophoresis, with a mutation detection sensitivity of 1%.
3. The single gene variation results in this report are based on evidence-based medicine annotations, that is, combining domestic and foreign databases and the center's self-built database for gene result annotations. This report only reports the variation frequency below 1% in each database and excludes benign variation sites.

Contents of blood disease-related fusion gene testing (71 genes)

ABL1	CRLF2	FGFR2	IL3	MECOM	NUP98	PRDM16	SETD2	TLX3
ABL2	CSFIR	FGFR3	JAK1	MEF2D	NUTM1	PTK2B	TP63	SFPQ
ALK	DUX4	FLT3	JAK2	MLLT10	P2RY8	RARA	STIL	TRDC
BCL11B	EP300	GATA2	JAK3	MYB	PAX5	RARB	TAL1	TYK2
BCR	EPOR	HLF	KMT2A	MYC	PBX1	RARG	TBL1XR	UBTF
CBFA2T3	ERG	HOXA9	LMO1	NPM1	PDGFRA	RBM15	TCF3	ZNF362
CBFB	ETV6	HOXA11	LMO2	NTRK3	PDGFRB	ROS1	TCRA	ZNF384
CREBBP	FGFR1	IKZF1	LYN	NUP214	PML	RUNX1	TLX	

Detection method

1. The total RNA of the sample was extracted by Trizol method, and then reverse transcribed into cDNA. The sequencing library was constructed with cDNA as template, and the sequencing was performed by Illumina NestSeq550 sequencing system.
2. The quality of all data was analyzed by bioinformatics to meet Q30>85%.
3. Fusion gene database: Fusion cancer, My Cancer Genome, Atlas of Genetics and Cytogenetics in Oncology and Haematology, etc.
4. Bioinformatics analysis of fusion gene method: STAR-fusion and other analysis software were used to predict fusion genes, and the reference genome was hg38.
5. This test project detects gene fusions within the coverage of the probe (see Appendix), and the detection sensitivity is about 0.1%.
