# Supplementary figures and images for: Case report: Identification of a novel TOR1AIP2::ETV6 transcript with FLT3-ITD mutation in acute myeloid leukemia progressed from myelodysplastic syndrome
Source: Front Oncol. 2024 Dec 10;14:1466590. doi: 10.3389/fonc.2024.1466590 (PMC11669196; doi:10.3389/fonc.2024.1466590)

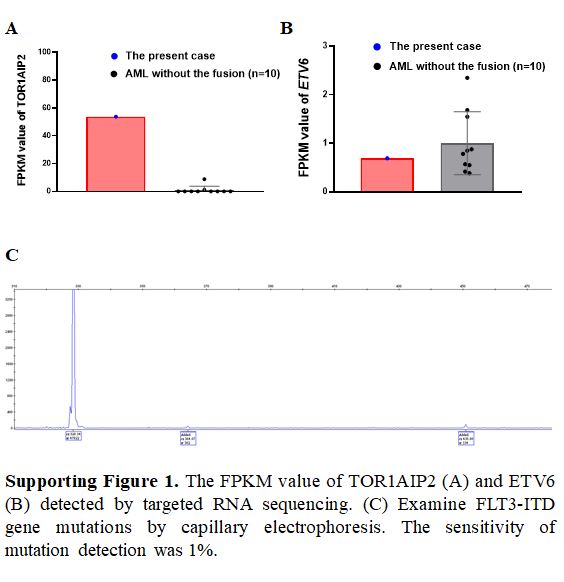

Supplement: Supplementary file 2 [file Image1.jpeg]
